# Diagnostic performance of the clear cell likelihood score integrated with cystic degeneration or necrosis on MR imaging for identifying clear cell renal cell carcinoma in cT1 solid renal masses

**DOI:** 10.1186/s13244-025-02029-y

**Published:** 2025-07-03

**Authors:** Xueyi Ning, Mengqiu Cui, Huiping Guo, Honghao Xu, Yuanhao Ma, Xu Bai, Shaopeng Zhou, Xiaohui Ding, Xiaojing Zhang, Huiyi Ye, Haiyi Wang

**Affiliations:** 1 Department of Radiology, The First Medical Center of the Chinese PLA General Hospital, Beijing, China; 2 Chinese PLA Medical School, Beijing, China; 3 Department of Pathology, The First Medical Center of the Chinese PLA General Hospital, Beijing, China

**Keywords:** Kidney neoplasms, Magnetic resonance imaging, Cystic degeneration, Necrosis, Diagnosis

## Abstract

**Objectives:**

To evaluate the diagnostic value of the clear cell likelihood score (ccLS) integrated with cystic degeneration or necrosis on renal MR imaging for diagnosing clear cell renal cell carcinoma (ccRCC) in cT1 solid renal masses (SRMs).

**Methods:**

This retrospective study consecutively enrolled patients with pathologically confirmed SRMs who underwent MRI at the First Medical Center of the Chinese PLA General Hospital between January 2022 and February 2024. Three radiologists independently scored all cT1 SRMs using ccLS and ccLS integrated with cystic degeneration or necrosis (cn-ccLS), with discrepancies reconciled by consensus. Sensitivity, specificity, and accuracy were used to assess the performance of ccLS and cn-ccLS.

**Results:**

A total of 287 patients with 293 masses were included in this study. The sample comprised 229 ccRCCs (78%), 64 other tumors. The sensitivity of cn-ccLS was significantly higher than ccLS (92% vs 74%; *p* < 0.001), with equal specificity to ccLS (88% vs 91%; *p* > 0.05). For cT1a and cT1b SRMs, the sensitivity of cn-ccLS was significantly higher than ccLS (cT1a: 90% vs 74%, *p* < 0.05; cT1b: 98% vs 75%, *p* < 0.001).

**Conclusions:**

Incorporating cystic degeneration or necrosis into the ccLS system significantly enhances the diagnostic performance of the ccLS system for ccRCC in cT1 SRMs. However, future validation of the ccLS system through large-sample, multi-center, and prospective studies is still required.

**Critical relevance statement:**

Incorporating cystic degeneration or necrosis into the ccLS system enhances performance for ccRCC in cT1 SRMs. It may enhance the value of ccLS and assist radiologists in their daily diagnostic work.

**Key Points:**

- The cn-ccLS effectively reduced the proportion of ccRCC among ccLS 3 lesions.
- cn-ccLS better diagnosed ccRCC for cT1a or cT1b renal masses than ccLS.
- ccRCC sensitivity was improved, but the impact on non-ccRCC remains unevaluated.

**Graphical Abstract:**

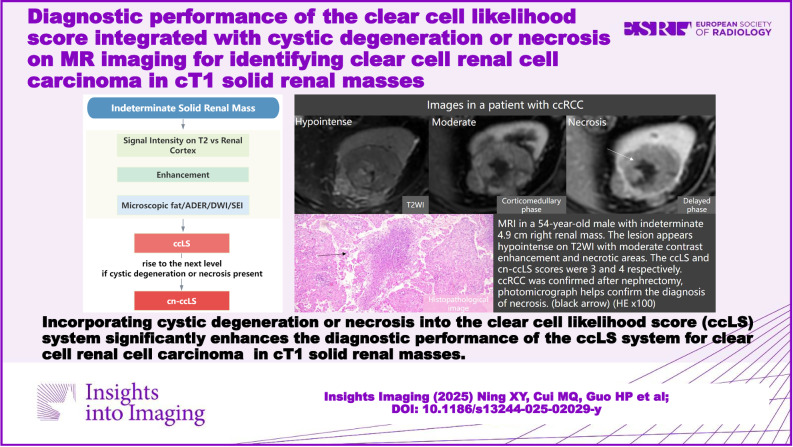

## Introduction

Renal tumors are one of the common urological tumors [1, 2]. The incidental detection of renal masses has increased due to advances in imaging technology [3–5]. Studies and guidelines [6–8] suggest that patients with cT1 tumors (the tumor is confined to the kidney, with a maximum diameter of ≤ 7 cm) usually have a relatively low risk of metastasis with an active surveillance strategy, although this risk varies by histologic subtype. Clear cell renal cell carcinoma (ccRCC), the most common malignant renal tumor subtype, accounts for 70–75% of renal cell carcinomas, has a poorer prognosis, and a higher risk of metastasis due to its aggressive nature [9–11]. Therefore, accurate preoperative characterization of ccRCC is particularly critical for determining subsequent treatment modalities.

Multiparametric magnetic resonance imaging (mpMRI) has proven valuable in diagnosing renal tumors [3, 12–15]. The clear cell likelihood score (ccLS) assigns a score to renal masses based on MRI indices to predict the likelihood of ccRCC [16, 17]. Simultaneously, the ccLS algorithm primarily identifies the three most common subtypes of RCC (ccRCC, chromophobe RCC, and papillary RCC) and the two most common benign renal tumors (fat-poor angiomyolipoma (fpAML) and renal oncocytoma (RO)), thereby providing a standardized classification of small renal masses [18, 19]. Several studies [20–23] have shown that ccLS has good diagnostic performance and moderate interobserver agreement for the diagnosis of ccRCC in cT1a and cT1b renal masses. However, there is still a risk of missed or misdiagnosed ccRCC, particularly with a score of 3 [20], which underscores the necessity of incorporating additional indices based on ccLS.

Cystic degeneration and necrosis are extremely prevalent in ccRCC, typically arising as a consequence of the tumor’s rapid growth and insufficient central blood supply [24–26]. It has been shown that necrosis can predict ccRCC and thus discriminate between fpAML and ccRCC [27]; it has also been shown that necrosis is usually absent within the RO [28]; in the cohort of Pedrosa et al, a trend towards less frequent cystic necrosis in chRCC and pRCC compared to ccRCC can be observed [29]. It has also been shown that ccRCC shows a higher proportion of cystic degeneration and necrosis than non-ccRCC [30]. This may suggest that cystic degeneration or necrosis on imaging has the potential to differentiate ccRCC from other tumors. However, the current ccLS system has not incorporated the two distinctive features, and it remains unclear what value these two imaging characteristics can contribute to the accurate diagnosis of ccRCC when integrated into the ccLS.

Furthermore, cystic degeneration or necrosis is manifested as non-enhancing areas within the tumor in clinical practice. However, significant overlap exists in cross-sectional imaging, which makes it difficult to identify cystic degeneration and necrosis. Therefore, this study aimed to investigate the diagnostic value of the ccLS scoring system in conjunction with cystic degeneration or necrosis for the accurate diagnosis of ccRCC of cT1 solid renal masses (SRMs).

## Materials and methods

### Patient cohort

This retrospective investigation received approval from the Ethics Committee of the First Medical Center of the Chinese PLA General Hospital (ethics no. S2024‑398‑02) with waived informed consent.

A radiologist (H.X.) consecutively searched the pathology database of our hospital for histologically confirmed renal neoplasms that were diagnosed between January 2022 and February 2024. All pathology specimens were obtained by surgical or percutaneous biopsy and assessed by a senior pathologist. The inclusion criteria were as follows: (a) cases with definite pathological results (five common renal tumor subtypes: ccRCC, chromophobe renal cell carcinoma (chRCC), papillary renal cell carcinoma (pRCC), fpAML, RO) obtained after MRI examination, no more than three months between MRI and surgery/percutaneous biopsy; (b) with complete MRI sequences; (c) solid renal mass: the solid component of renal mass > 25% on enhanced scans; (d) no macroscopic fat; (e) with a maximum tumor diameter of 1–7 cm; (f) the mass confined to the kidney; and (g) complete clinical data. The exclusion criteria were as follows: (a) treatment such as renal percutaneous biopsy and surgery before MRI examination; (b) poor-quality MRI images cannot be used for diagnosis and analysis, including motion artifacts or low image resolution; (c) infiltrative or non-circumscribed renal masses or metastases; (d) tumor invasion out of the kidney, lymph node metastasis, and distant organ metastasis; and (e) patients with three or more tumors simultaneously or renal masses associated with a known genetic syndrome (such as Von Hippel–Lindau or Birt–Hogg–Dubé).

### MRI protocol

The MR scans were performed on a GE Discovery 750 3.0-T scanner utilizing an 8-channel phased array coil, and the scanning range covered the entire kidney. The detailed MRI parameters can be found in the Supplemental Material.

### Imaging analysis and ccLS evaluation

**Fig. 1:**
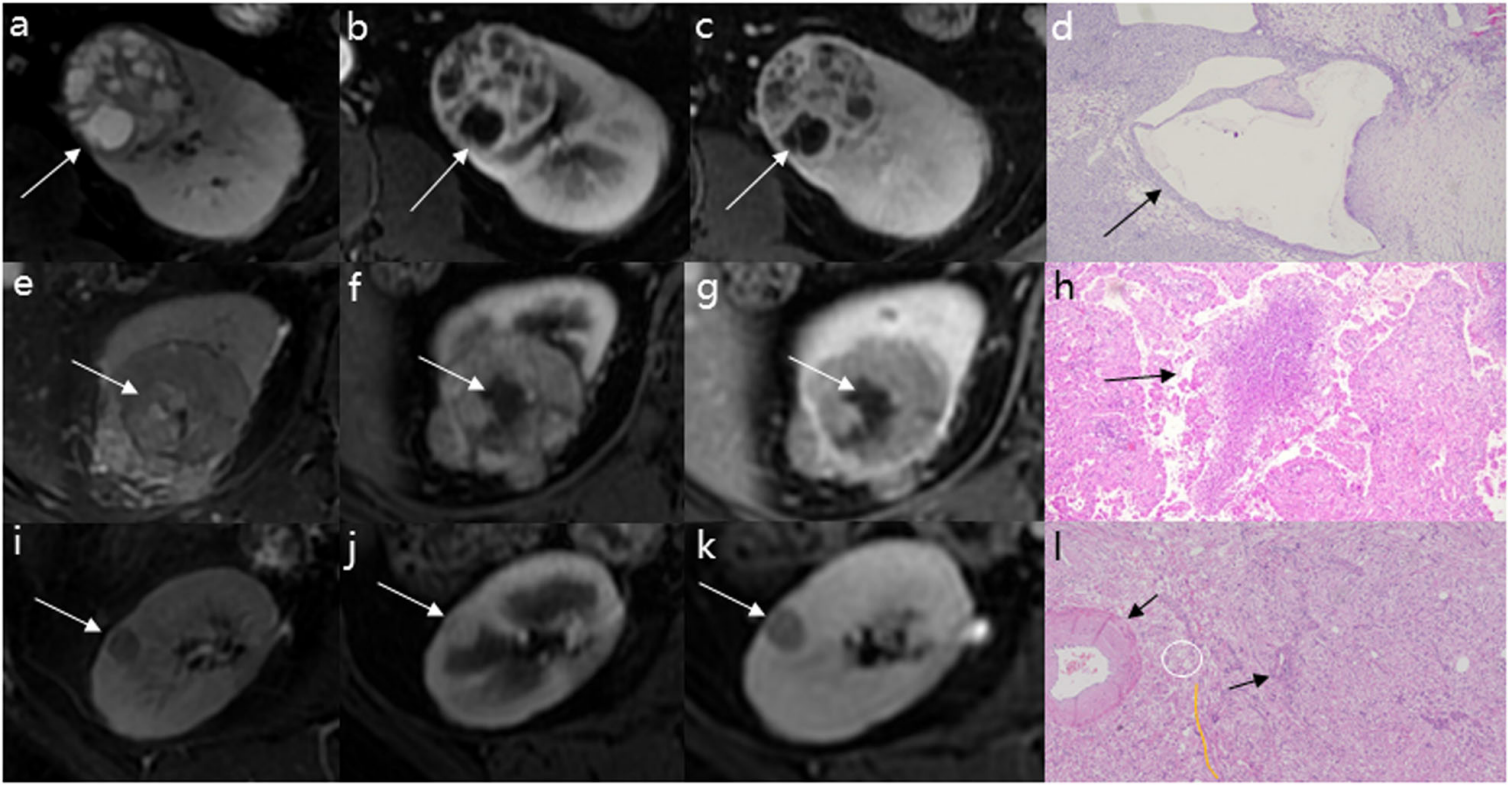
**a**–**d** MRI in a 44-year-old male with an indeterminate 4.7 cm left renal mass. The cystic generation in lesion shows hyperintense and regular shape (white arrow) on axial fat-saturated T2-weighted image (**a**), no enhancement (white arrow) on axial corticomedullary (**b**), and delayed phase (**c**) contrast-enhanced T1-weighted image, ccRCC was confirmed after nephrectomy, photomicrograph helps confirm the diagnosis of cystic degeneration (**d**) (black arrow) (HE ×100). **e**–**h** MRI in a 54-year-old male with an indeterminate 4.9 cm right renal mass. The necrosis in the lesion shows slightly hyperintense and irregular shape (white arrow) on axial fat-saturated T2-weighted image (**e**), no enhancement (white arrow) on axial corticomedullary (**f**), and delayed phase (**g**) contrast-enhanced T1-weighted image, ccRCC was confirmed after nephrectomy, photomicrograph helps confirm the diagnosis of necrosis (**h**) (black arrow) (HE ×100). **i**–**l** MRI in a 50-year-old female with an indeterminate 1.0 cm right renal mass. The lesion shows homogeneous and slightly hypointense regular mass (white arrow) on axial fat-saturated T2-weighted image (**i**), marked enhancement (white arrow) on axial corticomedullary (**j**), no cystic degeneration or necrosis on axial corticomedullary (**j**), and delayed phase (**g**) contrast-enhanced T1-weighted image, fat poor angiomyolipoma was confirmed after nephrectomy, photomicrograph helps confirm the diagnosis of blood vessels (black arrow), smooth muscle (yellow line), and fat (white circle) (**h**) (HE ×100)

**Fig. 2:**
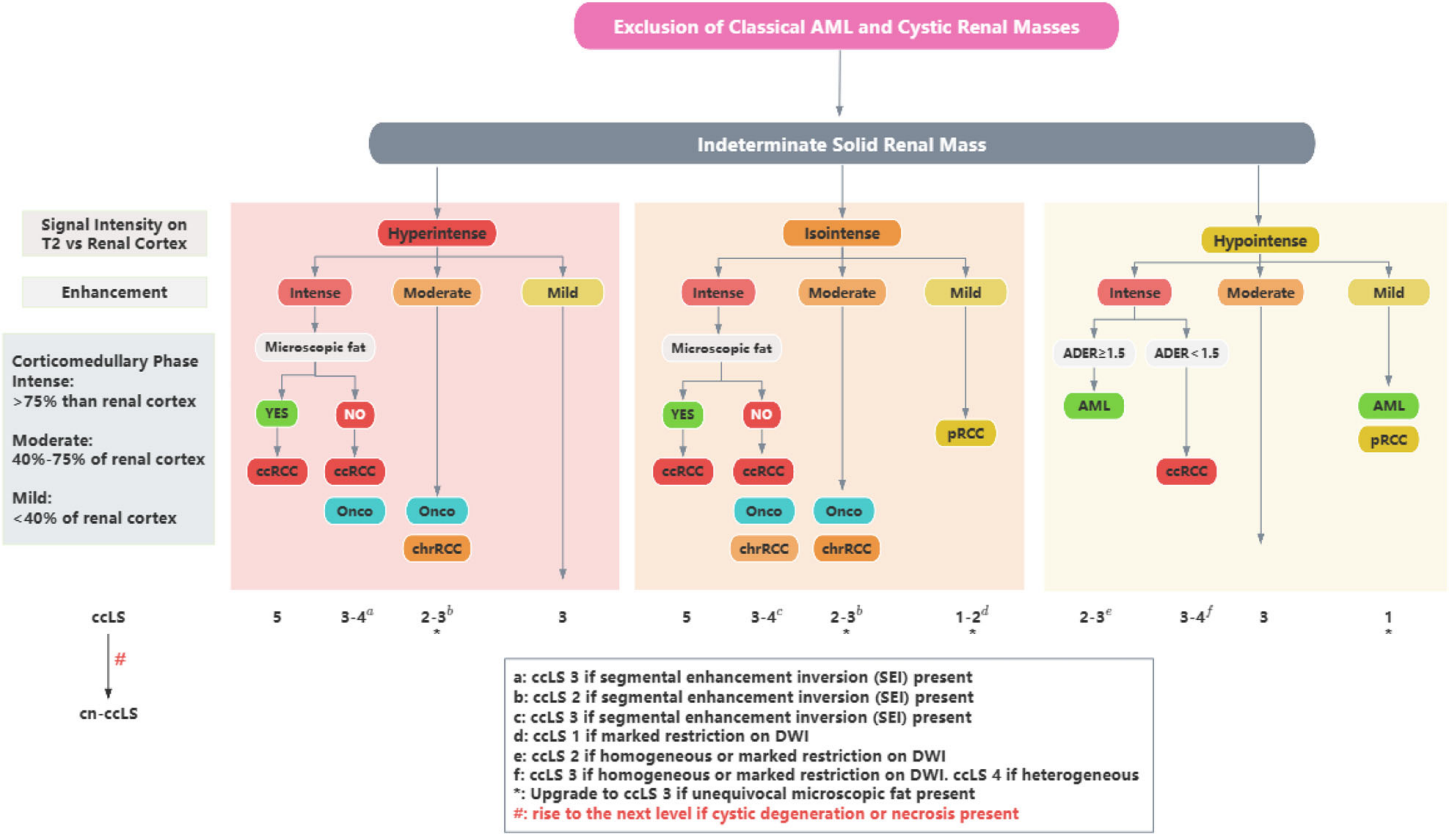
ccLS algorithm version 2.0 cn-ccLS. ccRCC, clear cell renal cell carcinoma; Onco, oncocytoma; chrRCC, chromophobe renal cell carcinoma; pRCC, papillary renal cell carcinoma; AML, angiomyolipoma; DWI, diffusion-weighted imaging [17]

Two radiologists (H.Y. and H.W.) with 37 years and 23 years of experience in abdominal diagnosis, respectively, provided comprehensive explanations and training to the evaluators regarding the detailed interpretation and scoring criteria of ccLS and the criteria for evaluation of cystic degeneration and necrosis. Three radiologists specializing in abdominal genitourinary imaging (X.N., H.G., and M.C. with 5 years, 6 years, and 15 years of experience, respectively) conducted the imaging analysis and ccLS evaluation independently, blinded to pathological results after undergoing training. The features to be assessed are listed below: (a) cystic degeneration: areas with high signal intensity (SI) on T2WI (similar to the fluid signal), low SI on T1WI, and lack of enhancement; (b) necrosis: high SI on T2WI (but not as high as fluid), low SI on T1WI, lack of enhancement, and a central location within the tumor. We take corticomedullary, nephrographic, and delayed phase images together to assess the unenhanced portion, and if none of the three phases are enhanced it represents a lack of enhancement; (c) indicators of ccLS: signal intensity at T2WI, corticomedullary enhancement, microscopic fat, restriction at DWI, segmental enhancement inversion (SEI), and arterial-delayed enhancement ratio; (d) ccLS: ccLS standardized the classification of SRMs by expressing the likelihood of renal clear cell carcinoma with five-tier probability scores, where 1 is very unlikely, 2 is unlikely, 3 is intermediate likelihood, 4 is likely, and 5 is very likely; and (e) ccLS integrated with cystic degeneration or necrosis (cn-ccLS): abbreviated as cn-ccLS, if cystic degeneration or necrosis is visible within the lesion, 1 point is added to the ccLS score and the ccLS 5 points remain unchanged. The detailed information of the features is shown in Table 1 and Fig. 1. The cn-ccLS flowchart is shown in Fig. 2.

**Table 1:** Specific definitions and notes for the required assessments

Assessment	Definition
1. Cystic degeneration	Areas with high SI on T2WI (similar to the fluid signal), low SI on T1WI, and a lack of enhancement
2. Necrosis	High SI on T2WI(but not as high as fluid), low SI on T1WI, lack of enhancement, and a central location within the tumor
Major criteria of ccLS [17]	
3. Signal intensity at T2WI	Predominant (> 50%) SI of the lesion at T2WI is characterized as hyper-, iso-, or hypointense compared with the renal cortex
4. Corticomedullary enhancement	a. Subjective diagnostic method: intense enhancement may be confidently made with a visual inspection, with renal masses enhancing as much as the renal cortex b. Quantitative diagnostic method: mild (PCE < 40%), moderate (PCE: 40–75%), or intense (PCE > 75%) enhancement relative to that of the renal cortex on the corticomedullary phase image
5. Microscopic fat	a. Subjective diagnostic method: a definitive decrease in SI on opposed-phase images compared with in-phase images at the same level is considered an unequivocal presence of microscopic fat in a renal tumor; the opposite proves that there is no microscopic fat b. Semi-quantitative diagnostic method: according to the formula, if (SI.tumor.IP − SI.tumor.OP) > (SD.IP − SD.OP), demonstrate the existence of microscopic fat. The opposite proves that there is no microscopic fat
Ancillary features of ccLS	
6. Restriction at DWI	If the predominant (> 50%) signal of the renal mass is higher on the DWI with a high (1000 mm^2^/s) *b* value and is lower on the ADC map compared with that of the renal cortex, then the renal mass is considered to have marked restriction
7. SEI	The presence of SEI is demonstrated if the portion of the lesion that enhances in the corticomedullary phase has a diminished signal in the delayed phase, whereas the portion of the lesion that does not enhance in the corticomedullary phase has an enhanced signal in the delayed phase
8. Arterial-delayed enhancement ratio	The formula is ADER = (SIart − SIpre)/(SIdel − SIpre); when ADER ≥ 1.5, it suggests the presence of a high amplitude of enhancement

*SI* signal intensity, *ccLS* clear cell likelihood score, *T2WI* T2-weighted imaging, *T1WI* T1-weighted imaging, *PCE* percent corticomedullary enhancement, *DWI* diffusion-weighted imaging, *ADC* apparent diffusion coefficient, *SEI* segmental enhancement inversion, *ADER* arterial-delayed enhancement ratio

### Pathological analysis

All pathological results of cT1 SRMs were reviewed by a pathologist with 25 years of experience according to the 2022 edition of the WHO Classification of Tumors of the Male Urological and Genitourinary System to exclude SRMs with ambiguous pathological findings.

### Statistical analysis

Statistical analysis was performed using STATA v17. The normality test was performed using the Shapiro–Wilk test, and data conforming to a normal distribution were expressed as mean ± standard deviation (SD), and data not normally distributed were expressed as median (interquartile range). Comparison of continuous variables between multiple groups was performed by ANOVA for data conforming to a normal distribution, Mann–Whitney *U*-test and Kruskal–Wallis method for data not conforming to a normal distribution, and chi-square test or Fisher’s exact probability method for categorical variables (such as sex, symptoms, and so on) depending on theoretical frequency.

Interobserver agreement was assessed for the above three readers’ assessments of overall ccLS/cn-ccLS, cystic degeneration, necrosis, and indicators of ccLS. Continuous variables (percent corticomedullary enhancement and arterial-delayed enhancement ratio) were assessed by the intraclass correlation coefficients (ICC) as follows: 0–0.50, poor; 0.50–0.74, moderate; 0.75–0.89, good; 0.90–1, excellent. Ordinal variables were assessed by Fleiss' kappa as follows: 0–0.20, poor agreement, 0.21–0.40, fair agreement, 0.40–0.60, moderate agreement, 0.61–0.80, substantial agreement, and 0.81–1.00, almost excellent agreement [31]. Differences in weighted kappa coefficients among the two score systems were compared using the Gwet agreement coefficient.

The final data used for analysis of sensitivity, specificity, and percentage of ccRCC in each category of ccLS and cn-ccLS were determined by the results in consensus among the above three radiologists (X.N., H.G., and M.C.). Diagnostic performance when ccLS and cn-ccLS were 4 or greater for diagnosing ccRCC was assessed by sensitivity, specificity, and accuracy using pathological findings as the gold standard (ccRCC and other tumors). The differences in sensitivity or specificity were compared using the McNemar test, and *p* < 0.05 was considered statistically significant.

## Results

### Patient and lesion characteristics

**Fig. 3:**
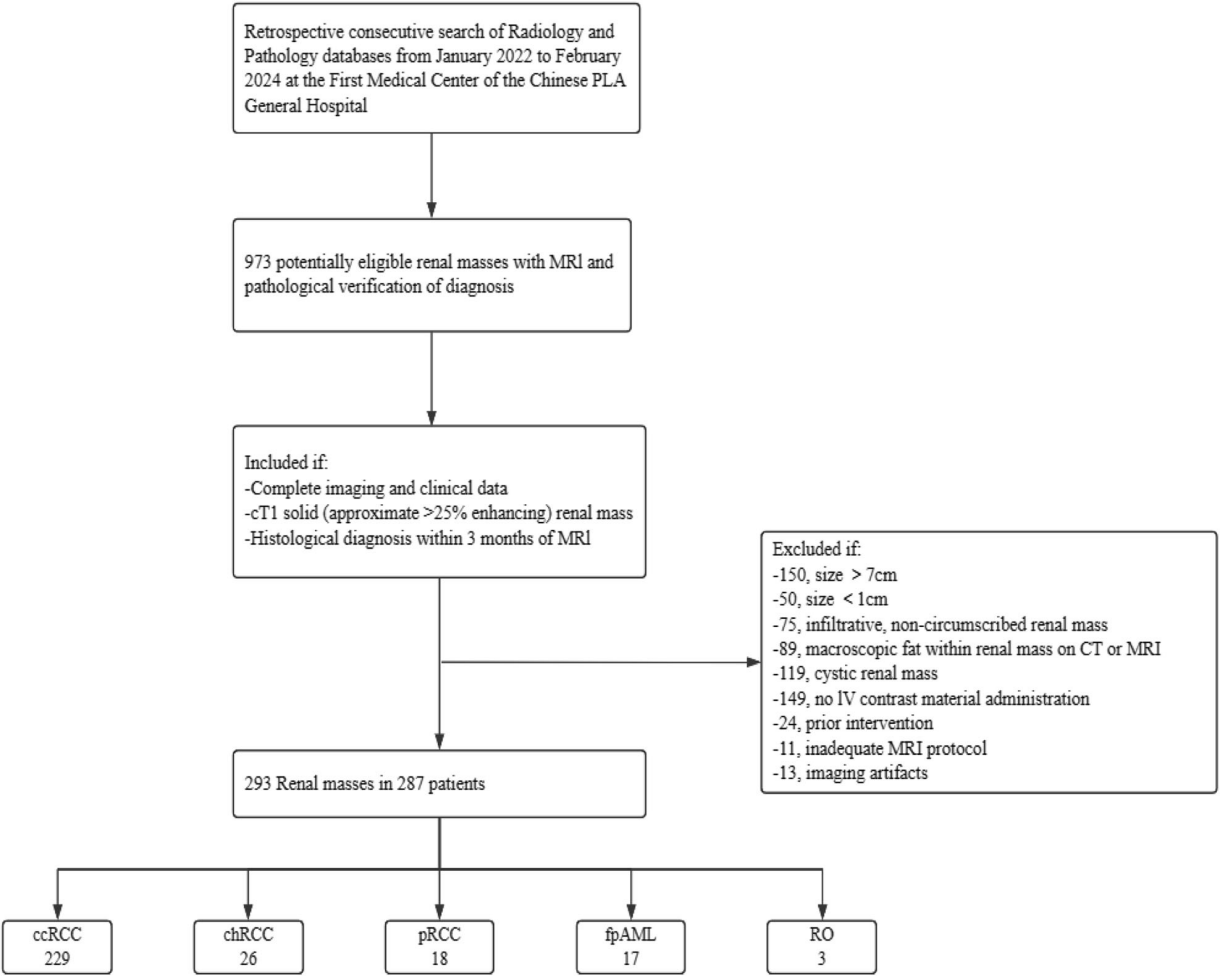
Flowchart for sample selection. ccRCC, clear cell renal cell carcinoma; chRCC, chromophobe renal cell carcinoma; pRCC, papillary renal cell carcinoma; fpAML, fat-poor angiomyolipoma; RO, renal oncocytoma

The flow diagram of patient selection is illustrated in Fig. 3. A total of 287 patients (192 males and 95 females; median age, 55 years; age range, 22–86 years) with 293 cT1 SRMs were included in the First Medical Center of the Chinese PLA General Hospital. 281 patients had single lesions, and 6 patients had two lesions. Among the 293 SRMs, 214 lesions were cT1a tumors, and 79 lesions were cT1b tumors. The age was 55 (46–63) years for patients with cT1a masses and 54.7 ± 11.4 (standard deviation) years for patients with T1b masses. The mean mass size was 2.9 (2.2–3.4) cm for cT1a masses and 4.9 (4.4–5.4) cm for cT1b masses.

Among all tumors, there were 229 ccRCCs (78%), 26 chRCCs (9%), 18 pRCCs (6%), 17 fpAMLs (6%), and 3 oncocytomas (1%). In the ccRCC group, there were 127 cases with cystic degeneration, 120 cases with necrosis, and 79 cases reclassified due to degeneration or necrosis. In the ccRCC group, there were 5 cases with cystic degeneration, 5 cases with necrosis, and 9 cases reclassified due to degeneration or necrosis. The detailed patient clinical data and tumor characteristics are shown in Table 2.

**Table 2:** Summary of patient and mass characteristics, stratified by histologic diagnosis

Characteristic	ccRCC (*n* = 229)	chRCC (*n* = 26)	pRCC (*n* = 18)	fpAML (*n* = 17)	RO (*n* = 3)	*p*
Histology total lesions^a^	229 (78)	26 (9)	18 (6)	17 (6)	3 (1)	
Age (y), M (IQR)	56 (46–64)	47.5 (37–55)	57.5 (52–65)	56 (46–60)	60 (55–64)	**0.027**
Size (cm), M (IQR)	3.3 (2.6–4.2)	3.2 (2.3–4.5)	3.1 (2.1–3.9)	2.8 (2.1–3.8)	2.4 (2.1–3.2)	0.370
BMI (kg/m²), M (IQR)	25.9 (23.4–28)	26.1 (23–29.2)	26.6 (24–29)	24.8 (23–26.6)	22 (22–30)	0.373
Sex^a^						**0.000**
Male	163 (71)	17 (65)	14 (78)	2 (12)	1 (33)	
Female	66 (29)	9 (35)	4 (22)	15 (88)	2 (67)	
Symptom^a^						0.992
Yes	11 (5)	1 (4)	1 (6)	1 (6)	0 (0)	
No	218 (95)	25 (96)	16 (94)	16 (94)	3 (100)	
Smoking history^a^	89 (39)	8 (31)	11 (61)	2 (12)	0 (0)	**0.021**
Drinking history^a^	126 (55)	15 (58)	7 (39)	1 (29)	2 (67)	0.194
Hypertension history^a^	92 (40)	6 (23)	10 (56)	7 (41)	1 (33)	0.289
Diabetes history^a^	35 (15)	1 (4)	3 (17)	0 (0)	0 (0)	0.196
Surgery^a^						0.639
PN	173 (76)	18 (69)	12 (67)	14 (82)	3 (100)	
RN	51 (22)	8 (31)	6 (33)	2 (12)	0 (0)	
MA	5 (2)	0 (0)	0 (0)	1 (6)	0 (0)	
T category^a^						0.560
cT1a	166 (72)	17 (65)	15 (83)	12 (71)	3 (100)	
cT1b	63 (28)	9 (35)	3 (18)	5 (29)	0 (0)	
Lesions with cystic degeneration^a^	127 (55)	2 (8)	4 (22)	0 (0)	0 (0)	0.000
Lesions with necrosis^a^	120 (96)	2 (2)	3 (2)	0 (0)	0 (0)	0.000
Cases reclassified due to cystic degeneration or necrosis^a^	79 (34)	4 (15)	5 (28)	0 (0)	0 (0)	0.000

*ccRCC* clear cell renal cell carcinoma, *chRCC* chromophobe renal cell carcinoma, *pRCC* papillary renal cell carcinoma, *fpAML* fat-poor angiomyolipoma, *RO* renal oncocytoma, *PN* partial nephrectomy, *RN* radical nephrectomy, *MA* microwave ablation, *BMI* body mass index

^a^ Case number (%)

*p* values with extremely significant differences are bolded

### Comparison of the diagnostic performance among ccLS and cn-ccLS

We analyzed the diagnostic performance of diagnosing ccRCC when ccLS or cn-ccLS was 4 or greater and compared the diagnostic performance among ccLS and cn-ccLS in the diagnosis of ccRCC in cT1 SRMs. When using ccLS, the sensitivity and specificity of three radiologists were 74% and 91% for all SRMs. When using cn-ccLS, the sensitivity and specificity of three radiologists were 92% and 88% for all SRMs (detailed results are shown in Table 3). Subgroup analyses were also performed according to different T-stages. In cT1a SRMs, the sensitivity of ccLS and cn-ccLS was 74% and 90%, respectively. In cT1b SRMs, the sensitivity of ccLS and cn-ccLS was 75% and 98%, respectively. We did not perform subgroup analyses for higher ccLS categories, as this was not feasible due to limitations in the number of cases.

**Table 3:** Comparison of diagnostic performance between different scoring systems in different stages of solid renal mass

T stage	Scoring system	Sensitivity (%)	p*	Specificity (%)	p*	PPV (%)	NPV (%)	Accuracy (%)
cT1a	ccLS	74		90		96	49	77
	cn-ccLS	90	**<** **0.001**	85	0.16	96	71	89
cT1b	ccLS	75		94		98	48	77
	cn-ccLS	98	**<** **0.001**	94	1	98	94	97
cT1	ccLS	74		91		97	49	77
	cn-ccLS	92	**<** **0.001**	88	0.16	96	76	91

*ccLS* clear cell likelihood score, *cn-ccLS* cystic degeneration or necrosis integrated with ccLS, *PPV* positive predictive value, *NPV* negative predictive value

* Compared with ccLS

*p* values with extremely significant differences are bolded

### Percentage of ccRCC in each category of ccLS and cn-ccLS

**Fig. 4:**
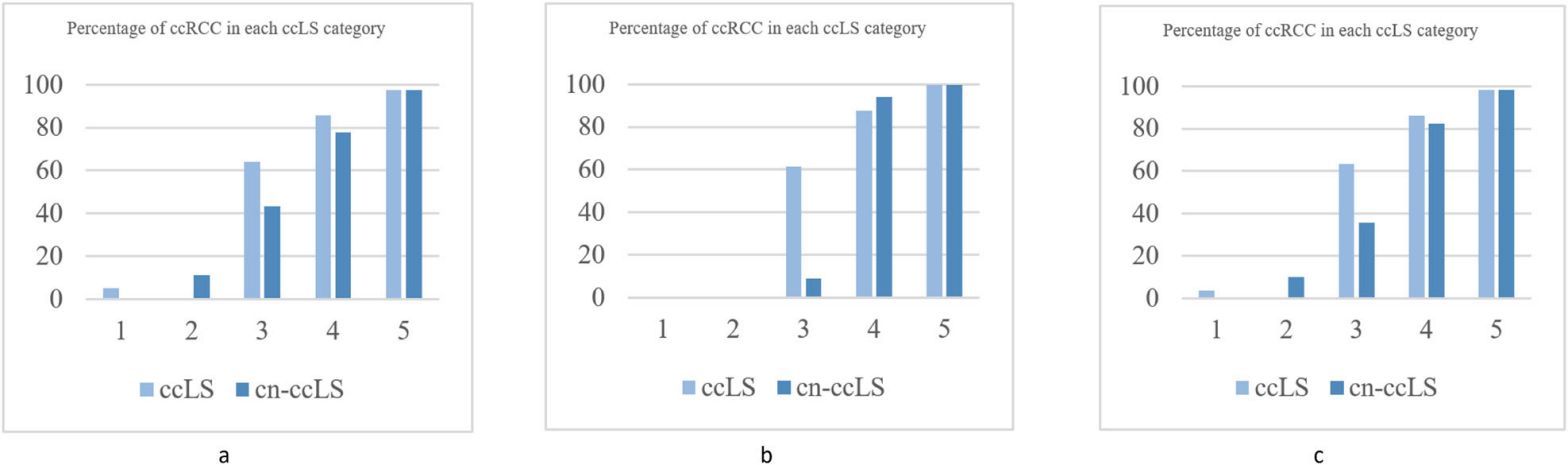
Percentage of ccRCC in each ccLS category using ccLS and cystic degeneration or necrosis integrated with ccLS (cn-ccLS) in cT1 (**a**), cT1a (**b**), and cT1b SRMs (**c**)

We counted the percentage of ccRCCs in each category of ccLS and cn-ccLS in different stages of SRMs (Fig. 4). It is noteworthy that the proportion of ccRCC in cn-ccLS 3-grade lesions is lower than that in ccLS 3-grade lesions. When ccLS and cn-ccLS were used for cT1 SRMs, the percentage of ccRCC corresponding to ccLS scores of 3 was 68% and 38%, respectively. With cT1a SRMs, the percentages were 68% and 46%, respectively. With cT1b SRMs, the percentages were 64% and 10%, respectively.

### Interobserver agreement

The κ values of cystic degeneration, necrosis, and cystic degeneration or necrosis were 0.64 (95% CI: 0.51–&&0.76), 0.49 (95% CI: 0.36–0.62), and 0.76 (95% CI: 0.65–0.88). We also performed a consistency analysis of the ccLS metrics. The κ values of T2WI signal intensity, microscopic fat, and SEI were 0.53 (95% CI: 0.44–0.61), 0.39 (95% CI: 0.23–0.54), and 0.17 (95% CI: 0.00–0.33). The ICC values of percent corticomedullary enhancement and arterial-to-delayed enhancement ratio were 0.70 (95% CI: 0.60–0.80) and 0.46 (95% CI: 0.34–0.58).

We compared the interobserver agreement between cn-ccLS and ccLS in different stages of SRMs (Table 4). In all SRMs, the κ values of ccLS and cn-ccLS were 0.62 (95% CI: 0.44–0.65) and 0.71 (95% CI: 0.60–0.82), respectively. In cT1a SRMs, the κ values of ccLS and cn-ccLS were 0.62 (95% CI: 0.50–0.75) and 0.70 (95% CI: 0.58–0.83), respectively. In cT1b SRMs, the κ values for ccLS and cn-ccLS were 0.56 (95% CI: 0.34–0.77) and 0.70 (95% CI: 0.50–0.91), respectively.

**Table 4:** Comparison of interobserver agreement between different scoring systems in different stages of solid renal mass

T stage	κ		*p*
	ccLS	cn-ccLS	
cT1a	0.62 (0.50, 0.75)	0.70 (0.58, 0.83)	**<** **0.001**
cT1b	0.56 (0.34, 0.77)	0.70 (0.50, 0.91)	**<** **0.001**
cT1	0.62 (0.44, 0.65)	0.71 (0.60, 0.82)	**<** **0.001**

Data in parentheses represent 95% CI

*ccLS* clear cell likelihood score, *cn-ccLS* cystic degeneration or necrosis integrated with ccLS, *CI* confidence interval

*p* values with extremely significant differences are bolded

## Discussion

In this study, we incorporated cystic degeneration or necrosis into the ccLS framework and compared the diagnostic performance of ccLS and cn-ccLS using data from 287 SRM patients (293 tumors) from a single center. Compared to ccLS, the cn-ccLS demonstrated superior performance; it exhibited higher sensitivity than ccLS while maintaining equivalent specificity, thus confirming the added value of integrating cystic degeneration and necrosis.

Consistent with the findings of previous studies [27, 30, 32], our study also revealed that cystic degeneration and necrosis are more frequently associated with ccRCC than other tumor subtypes. This may be related to the pathogenesis and pathological mechanisms of ccRCC. Characterized by heightened intratumoral hypoxia and elevated expression of angiogenic genes, ccRCC tends to grow more rapidly than other tumors [24–26]. This accelerated growth increases the likelihood of secondary hemorrhage, necrosis, and additional complications within the tumor, ultimately fostering the development of cystic degeneration [33–35]. This may suggest that cystic degeneration or necrosis on imaging has the potential to differentiate ccRCC from other tumors.

Based on previous research and clinical experience [36], we discovered that cystic degeneration and necrosis can be challenging to distinguish on MRI in clinical practice, especially for junior observers. Therefore, when we established the specific criteria for cn-ccLS, we assumed that if cystic degeneration or necrosis was present within the lesion, an additional point would be added to the original score. By comparing the diagnostic performance of ccLS and cn-ccLS, we observed improved diagnostic efficacy after integrating cystic degeneration or necrosis into ccLS, with the sensitivity increasing from 74% to 92%.

In addition to analyzing the diagnostic performance of all lesions, we conducted a subgroup analysis by dividing all tumors into cT1a and cT1b groups according to the T stage. The results showed that the diagnostic performance of cn-ccLS was significantly improved compared with ccLS in both cT1a and cT1b groups, with the sensitivity improving from 74% to 90% and from 75% to 98%, respectively. The accuracy of our cn-ccLS is numerically higher than the accuracy of ccLS reported by Dunn et al [22], both in the cT1a and cT1b SRMs (89% vs 83% in cT1a SRMs, 97% vs 92% in cT1b SRMs). The cn-ccLS performs better in cT1b renal masses. This improvement may be attributed to the higher incidence of cystic degeneration and necrosis observed in tumors larger than 4 cm in our study (76% vs 63%). Insufficient blood supply in the center of rapidly growing tumors leads to cystic degeneration and necrosis within the tumors. Liu et al [37] showed a correlation between larger tumor size and the presence of cystic degeneration and necrosis, which is consistent with the findings of this study.

Previous studies [17, 38] have identified a significant proportion of ccRCC cases within ccLS 3 lesions in ccLS, indicating potential underdiagnosis. Our study observed a similar phenomenon, with ccLS 3 lesions comprising 30% of the cases, of which 67.8% were ccRCC. We found that cn-ccLS successfully reduced the percentage of ccRCC within ccLS 3 lesions, decreasing the percentage of ccRCC from 68% to 38% in the original ccLS 3 lesions. Concurrently, the sensitivity of cn-ccLS was significantly higher than ccLS’s (74% vs 92%, *p* < 0.001). The cn-ccLS effectively mitigates the risk of underdiagnosing ccRCCs. A potential explanation for this improvement lies in the observation that up to 71.2% of ccRCC demonstrated cystic degeneration or necrosis areas in ccLS 3 lesions. If a ccLS 3-point lesion exhibits cystic degeneration or necrosis, it is escalated to ccLS 4, thereby enhancing the diagnostic system’s sensitivity.

The ccLS assigns scores to renal tumors based on the radiologists’ subjective assessment, rendering inter-observer variability inevitable. In line with previous findings [22], our study’s kappa value of ccLS is 0.62. Compared to ccLS, the inter-observer agreement of cn-ccLS was significantly improved (0.71 vs 0.62). This may be attributed to the rules of cn-ccLS. The presence of either cystic degeneration or necrosis adds one point to the ccLS. Notably, the agreement of cystic degeneration and necrosis was superior to most of the original subjective indicators of ccLS in this study, such as microscopic fat and SEI (κ = 0.76 for cystic degeneration or necrosis vs 0.39 for microscopic fat, 0.17 for SEI). Moreover, incorporating cystic degeneration or necrosis into ccLS does not alter the original pathway. Consequently, the agreement of cn-ccLS significantly outperformed ccLS. The combination of these two features could improve the diagnostic performance of ccLS and enhance the diagnostic confidence of radiologists.

### Limitations

This study has some limitations. Firstly, it is a retrospective single-center study, and only five common renal tumors identified by the ccLS algorithm were included in this study, which inevitably leads to a certain degree of selection bias. In the future, an attempt could be made to include all pathology types and perform a multicenter validation of cn-ccLS. Secondly, this study exclusively included renal tumors with confirmed pathology, excluding cases confirmed by imaging features and follow-up, which may also contribute to selection bias. Thirdly, this study defined cystic degeneration and necrosis signs as imaging indicators on magnetic resonance images based on prior experience and literature. The imaging-pathological correspondence of cystic necrosis signs can be further explored in the future. The varying expertise of different observers and their interpretations of these signs may impact the application of cn-ccLS in clinical practice. Finally, the high proportion of ccRCC in this study may affect NPV and PPV. Validating after matching the number of positive cases with the number of negative cases might be helpful.

## Conclusion

In summary, this MRI-based study demonstrates that the incorporation of cystic degeneration and necrosis into the ccLS system significantly enhanced its diagnostic performance for ccRCC in cT1 SRMs. It may enhance the clinical application value of ccLS, and assist radiologists in their daily diagnostic work. However, large samples, multi-center, prospective data are still needed to validate them in the future.

## Supplementary information

ELECTRONIC SUPPLEMENTARY MATERIAL

## Data Availability

The datasets used and/or analyzed during the current study are available from the corresponding author on reasonable request.

## Abbreviations

ccLS: Clear cell likelihood score
ccRCC: Clear cell renal cell carcinoma
chRCC: Chromophobe renal cell carcinoma
cn-ccLS: ccLS integrated with cystic degeneration or necrosis
fpAML: Fat-poor angiomyolipoma
pRCC: Papillary renal cell carcinoma
RO: Renal oncocytoma
SEI: Segmental enhancement inversion
SRMs: Solid renal masses
