# Supplementary material for: Diagnostic performance of the clear cell likelihood score integrated with cystic degeneration or necrosis on MR imaging for identifying clear cell renal cell carcinoma in cT1 solid renal masses
Source: Insights Imaging. 2025 Jul 3;16:149. doi: 10.1186/s13244-025-02029-y (PMC12229434; doi:10.1186/s13244-025-02029-y)
Supplement: Supplementary file 1 — ELECTRONIC SUPPLEMENTARY MATERIAL [file 13244_2025_2029_MOESM1_ESM.pdf]

Diagnostic Performance of the Clear Cell Likelihood Score Integrated with Cystic Degeneration or Necrosis on MR Imaging for Identifying Clear Cell Renal Cell Carcinoma in cT1 Solid Renal Masses

ELECTRONIC SUPPLEMENTARY MATERIAL

MRI Protocol

The minimum MRI requirements included: (a) an axial and/or coronal T2-weighted imaging with or without fat suppression (fast spin echo or single shot fast spin echo sequence was used); (b) an axial single-shot echo-planar diffusion-weighted imaging with low (50 mm2/sec) and high (1000 mm2/sec) b values; (c) a dual-echo chemical shift (in- and opposed-phase) T1-weighted gradient-recalled echo imaging; (d) pre- and dynamic postcontrast T1-weighted sequences with fat suppression: three-dimensional fast spoiled gradient echo sequence was used, including the corticomedullary phases (approximately 30-40 seconds), nephrographic phases (approximately 90-120 seconds) and delayed phases (approximately 240-360 seconds). Gadobenate dimeglumine (Shanghai Bracco Sine Pharmaceutical Co., Ltd., China) was injected intravenously at a dose of 0.1 mmol/kg from a high-pressure syringe at a flow rate of 1.5 ml/s, and the 20 ml of saline was injected at the same flow

TABLE S1: **Specific Notes for the Required Assessments**

Assessment	Note
1. Cystic degeneration	
2. Necrosis	
**Major criteria of ccLS**	
3. Signal intensity at T2WI	a. Fat-suppressed T2WI is also available<br>b. Hemorrhage, necrosis, cystic degeneration, and tumor margins should be avoided<br>c. Only the SI of the solid part of the lesion should be evaluated
4. Corticomedullary enhancement	a. Differentiation between mild and moderate often requires quantitative analysis using an ROI, the area of ROI is approximately 100 mm^2<br>b. Place the ROI in the area with the most obvious enhancement on the corticomedullary phase image, place the same ROI (with the same position and size) on the corresponding pre-contrast image, and place it on the renal cortex in the same way as above, and measure the SI and calculate the percentage of enhancement respectively.<br>c. Calculation formula: PCE=[(TCM-TPre)/TPre]/[(CCM-CPre)/CPre] ×100%.<br>d. TCM and CCM denote the SI of the lesion and renal cortical corticomedullary phase, respectively; and TPre and CPre are the SI of the lesion and renal cortex on the pre-contrast T1WI
5. Microscopic fat	a. When the presence of microscopic fat cannot be determined by visual inspection, we can use the semi-quantitative formula<br>b. We can place the ROIs in the suspected areas containing microscopic fat of the in-and opposed-phase, and record values of SI.tumor.IP, SI.tumor.OP, SD.IP, SD.OP.<br>c. SI.tumor.IP and SI.tumor.OP denotes the average SI of the lesion on the in- and opposed-phase, respectively; SD.IP and SD.OP denotes the standard deviation of the SI of the lesion on the in- and opposed-phase images, respectively
**Ancillary features of ccLS**	
6. Restriction at DWI	

7. Segmental enhancement inversion	
8. Arterial-delayed enhancement ratio	a. SIart, SIpre, and SIdel are the signal intensities of corticomedullary, pre-contrast, and delayed phases of the lesion, respectively

SI=signal intensity; ccLS=clear cell likelihood score; T2WI= T2-weighted imaging; T1WI= T1-weighted imaging; PCE= percent corticomedullary enhancement; DWI= diffusion weighted imaging; ADC=apparent diffusion coefficient; SEI= segmental enhancement inversion; ADER=arterial-delayed enhancement ratio
